# Wireless Sensor Networks for Big Data Systems

**DOI:** 10.3390/s19071565

**Published:** 2019-04-01

**Authors:** Beom-Su Kim, Ki-Il Kim, Babar Shah, Francis Chow, Kyong Hoon Kim

**Affiliations:** 1 Department of Computer Science and Engineering, Chungnam National University, Daejeon 34134, Korea; bumsou10@naver.com or bumsou10@cnu.ac.kr; 2 College of Technological Innovation, Zayed University, Abu Dhabi 144534, UAE; babar.shah@zu.ac.ae; 3 University College, Zayed University, Abu Dhabi 144534, UAE; Francis.Chow@zu.ac.ae; 4 Department of Informatics, Gyeongsang National University, Jinju 52828, Korea; khkim@gnu.ac.kr

**Keywords:** wireless sensor networks, big data, infrastructure, data processing

## Abstract

Before discovering meaningful knowledge from big data systems, it is first necessary to build a data-gathering infrastructure. Among many feasible data sources, wireless sensor networks (WSNs) are rich big data sources: a large amount of data is generated by various sensor nodes in large-scale networks. However, unlike typical wireless networks, WSNs have serious deficiencies in terms of data reliability and communication owing to the limited capabilities of the nodes. Moreover, a considerable amount of sensed data are of no interest, meaningless, and redundant when a large number of sensor nodes is densely deployed. Many studies address the existing problems and propose methods to overcome the limitations when constructing big data systems with WSN. However, a published paper that provides deep insight into this research area remains lacking. To address this gap in the literature, we present a comprehensive survey that investigates state-of-the-art research work on introducing WSN in big data systems. Potential applications and technical challenges of networks and infrastructure are presented and explained in accordance with the research areas and objectives. Finally, open issues are presented to discuss promising directions for further research.

## 1. Introduction

When constructing a big data system, data collection, storage, processing, analysis, and visualization are steps that need to be followed in the said order. In ongoing research on big data systems, the research communities focus on fundamental aspects of dealing with big data: specific platforms, technology, beneficial applications, standards, and best practices (for applications in social web, financial issues, and so on). Moreover, there are many platforms and tools that can implement these functions in the real world. Thus, data-intensive applications are now being developed to benefit from them. Chen et al. [1] present state-of-the-art technologies to deal with the problems of big data systems (specifically, computing infrastructure for data deluge that includes granular computing, cloud computing, bio-inspired computing, and quantum computing). Moreover, seven principles to be applied in big data systems are defined and explained. In addition, Chen et al. [2] provided a general overview of big data systems, focusing on the four phases of value chain of big data. In their survey, they discuss the technical challenges in data generation, acquisition, storage, and analysis as separate phases. Moreover, a new survey of big data was recently published by Oussous et al. [3], which addresses new data techniques and platforms, with the aim to select a right combination of technologies according to the technological needs and the specific applications requirements.

As reported in the aforementioned studies, data collection is the first step of building a big data system. Among the many available data-generating sources, wireless sensor networks (WSNs) are receiving considerable research attention with regard to environmental monitoring. A WSN consists of a large number of sensor nodes that monitor and record the physical conditions of an environment, and the sensor data are collected at a so-called sink node. WSNs are used to measure environmental conditions: temperature, sound, pollution levels, humidity, wind, and so on. However, the limited capacity of a single node and a narrow wireless link (compared to typical networks) cause problems with delivering the sensor data to the sink node. Nevertheless, an effective data aggregation and in-network processing are beneficial to big data systems. Therefore, there is a need of analyzing research studies that link WSN and big data systems while overcoming the deficiencies of WSN and improving system performance.

**Figure 1:**
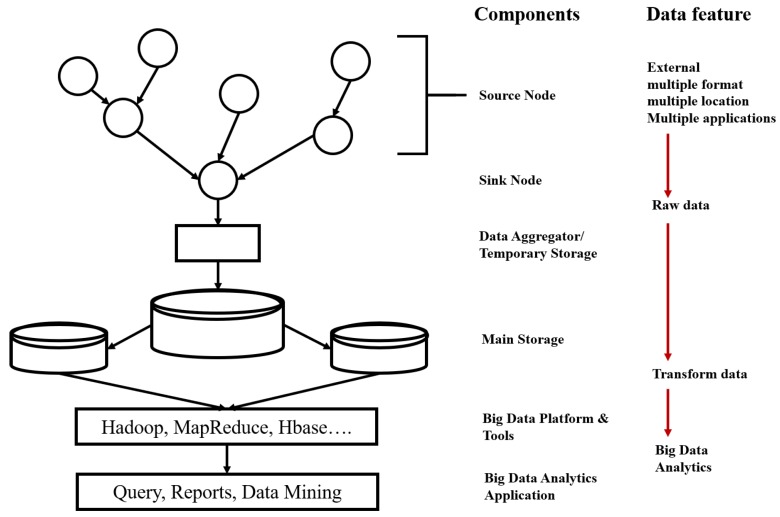
Big data system based on a WSN.

Figure 1 illustrates a basic structure of a big data system based on a WSN as an example of a fundamental system architecture. As shown in Figure 1, a sink node collects the data from sensor nodes and then delivers the data to a temporary storage for consequent data aggregation. After this step, the aggregated data can be manipulated by a big data framework using the main storage. The transformed data are handled by big data platforms and applications. Special issues of journals and many other papers have been published mechanisms to integrate a WSN as the main source in a big data system using the abovementioned architecture. An example of the special issue is “Big Data in Ubiquitous Wireless Sensor Networks” edited by Xiao et al. [4]; in this issue, 18 papers are presented to show advances of big data systems with ubiquitous WSNs in terms of computation and storage over cloud computing. Moreover, technical issues for big data in three-dimensional underwater sensor networks, wireless video sensor networks, wireless multimedia sensor networks, and typical WSNs are explained and described. In a comprehensive survey, Halde et al. [5] presented the issues and challenges of data collection through WSNs. The authors focused on energy consumption and heterogeneity of WSNs, which are observed during the analysis of big data in WSN. Harb et al. [6] addressed data management issues in WSNs by introducing different algorithms designed for data collection, aggregation, correlation, compression, and prediction; in addition, they showed potential and feasible application scenarios.

**Figure 2:**
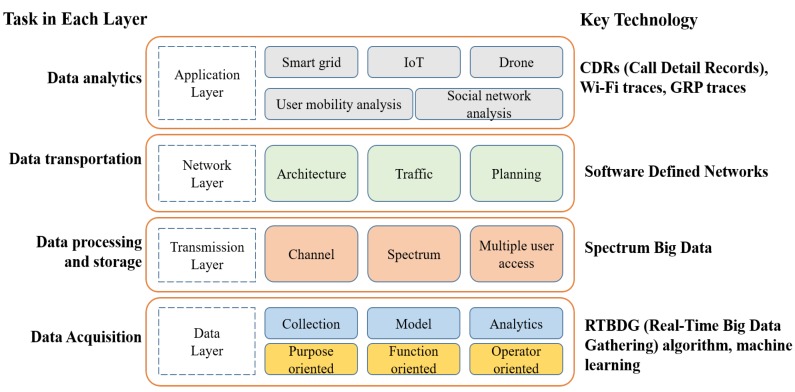
Protocol layering of wireless big data systems.

Convergence problems between WSNs/internet-of-things (IoT) and big data are reviewed and analyzed in [7], wherein the following open issues are mentioned: convergence process, security, management of data, interoperability, and hardware/architecture challenges. From the point of view of data usage, data collected from IoT contribute to context-aware computing, such as ubiquitous and pervasive computing. Thus, big data issues in ambient intelligence and WSNs need to be explored well. To achieve this goal, Sezer et al. [8] present open issues and provide an insight on IoT for big data. Furthermore, a wireless big data system that deals with massive data in wireless networks is described in [9]. Instead of describing the entire system, the authors focused on wireless infrastructure such as data-aided transmission, data-driven network optimization, and novel applications under layered architecture (as shown in application, network, transmission, and data layers in Figure 2). Further, they discussed three potential application areas: smart grids, internet-of-things (IoT), and drones/unmanned aerial vehicles (UAV), as illustrated in Figure 2.

Despite the aforementioned research efforts, a comprehensive survey that provides deep insight into most research areas for WSNs as a source for big data remains lacking. To address this gap in the literature, in this paper, we present a comprehensive survey of research that introduces WSNs as a source of big data. In order to analyze the state-of-the-art for this research area, only the most recent papers (published in 2014 or later) addressing WSNs and big data simultaneously have been selected. Even though many existing research studies are applicable to big data systems, not all of them are included here because we focus on only integrated systems. Based on this selection strategy, we describe the potential applications of big data systems based on WSNs in Section 2. Then, we categorize the current research work according to the research areas in Section 3. Open issues in these research areas are introduced and discussed in Section 4. Finally, we conclude the paper in Section 5.

## 2. Applications of WSN-Based Big Data Systems

Before starting a detailed analysis of related work, it is highly desirable to understand which big data applications can be implemented and deployed through WSNs. Since a WSN is usually built to meet application-specific requirements, it is reasonable to review big data applications prior to addressing their technical issues.

The following monitoring applications can benefit from using WSNs: smart grids, monitoring human body, and monitoring the environment.

In case of smart grids [10], smart sensor networks are introduced in big data systems for energy management. These systems run smart grid applications that include power monitoring, demand-side energy management, coordination of distributed storage, and integration of renewable energy generators. Additionally, techniques used to manage big data generated by sensors and meters are proposed. Moreover, feasible recommendations and practices for smart grid are discussed. The authors in [11] focus on managing data efficiently and extracting required information from the big data system. Because reliability and low latency are two objectives in a smart grid, streaming processing on fog computing architecture is studied for real-time applications over a well-designed platform.

**Figure 3:**
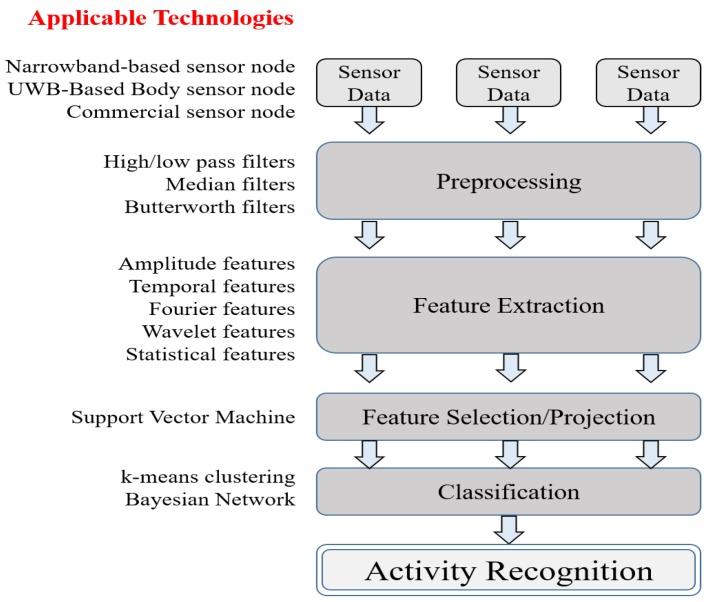
Typical activity recognition process for inferring activities from WBAN sensors.

The next example is monitoring the human body: wireless body area networks (WBAN). Collection of a vast amount of health and medical data via body sensor networks is presented for big data systems in [12]. To implement body sensor networks, an activity recognition application is required to implement the following functionality: feature extraction and selection, classification, supporting software platforms, and sensor and user authentication. This typical activity recognition procedure is classified and illustrated in Figure 3, which describes the general steps in [12]. Du et al. [13] present a feasible WBAN big data framework, which uses MapReduce for processing big data in real time, and Hadoop/HBase for storing and analyzing WBAN’s big data. Another interesting application of monitoring abnormal conditions of heart rate is proposed by Yuan et al. [14], with ZigBee and big data analysis based pulse monitoring system. To avoid missing the pulse signal, two following methods were proposed: (1) the photo-electricity based dynamic continuous heart rate monitoring methods, and (2) comprehensive anti-jamming methods. Using these two methods, a training model based on big data is proposed to improve the physical training level and to create training plans. With regard to data classification and detecting atypical events (anomaly detection), Wu et al. [15] proposed a new feature-based learning system for IoT applications that is based on the theory of distributed compression as well as the sparsity and relativity of data.

Next, we consider examples of environmental monitoring, such as big data systems that monitor air quality in industrial workplace buildings. A case study of a big data system that monitors air quality collected by WSN is presented in [16]. Locations include two workshops that are part of a large on-shore logistics base of a regional shipping industry in Norway. The study is conducted to prove the efficiency of data analytics and visualization. Substantially, the case study reveals the possibility to monitor worker safety in other high-risk industries, as well as the quality of goods in supply chain management by integrating WSNs and big data systems. Moreover, as a possible monitoring application, cooperative fire security system using human agent robot machine sensor (CFS${}^{2}$H) message protocol [17] for a firefight system is presented to provide fast communication and stable collaboration. The stationary WSN node is responsible of generating the data, while a big data center controls the whole system’s work in the suggested architecture.

At last, big data systems for smart cities are proposed in [18] to find feasible solutions for public administration, environment, and urban services. In this analysis, the data is converged into a shape similar to a map (that could be paper maps or digital ones) of e-government services. Usually, e-government services are known to improve efficiency and citizen satisfaction due to their implementation of spatial data infrastructure (SDI). Therefore, it is proven that e-government based on big data and WSN improves the access to e-services. Moreover, it could be used to overcome challenges of managing limited resources.

This concludes our analysis of applications. In the next section, we present the technical approaches.

## 3. Technical Approaches to WSN-Based Big Data Systems

### 3.1. Big Data Through WSN

Data generated by the sensors grow exponentially. Conventional information technologies for data processing, storage, and reporting (such as servers and relational databases) are too expensive to deal with these data. Moreover, they cannot cope with the processing needs that can be required for real-time processes. In addition, most of the events monitored at regular intervals are largely redundant or are minor variations leading to a large waste of data storage resources and communication energy at relay and sensor nodes. This implies that much of these data are of no interest, meaningless, and redundant. Thus, unlike the case of typical WSNs, it is essential to gather and transmit a large amount of data while minimizing data latency in WSN-based big data systems. Moreover, it is required to efficiently eliminate data redundancy and improve energy efficiency. The overlap between big data systems and WSNs lies in the use of in-network data processing techniques, as indicated in [19]. For the WSNs side, it would save their limited resources. At the same time, receiving a clean, non-redundant, and relevant data would reduce the excessive data volume at the side of the big data system. Thus, it would reduce overload by discovering values from these data rapidly.

The research challenges for WSN-based big data systems are addressed in [20,21]. Boubiche et al. [20] classified big data challenges in wireless sensor networks into clustering, securing, processing, and energy saving. The proposed strategies of dealing with these challenges are based on the correlation between the data aggregation, clustering, and energy consumption challenges of big sensor data. Djedouboum et al. [21] described big data collection in a large-scale WSN, presented data transferring scheme in the context of large-scale WSN, and discussed the challenges of big data collection. An example architecture for gathering, storage, and analysis of data generated by WSN for monitoring air pollution levels in a city was proposed as a prototype in [22]. The prototype was developed with open source technologies such as Storm for stream processing, Hadoop for distributed computing with MapReduce, and a small WSN on the Arduino platform.

### 3.2. Categorization

**Figure 4:**
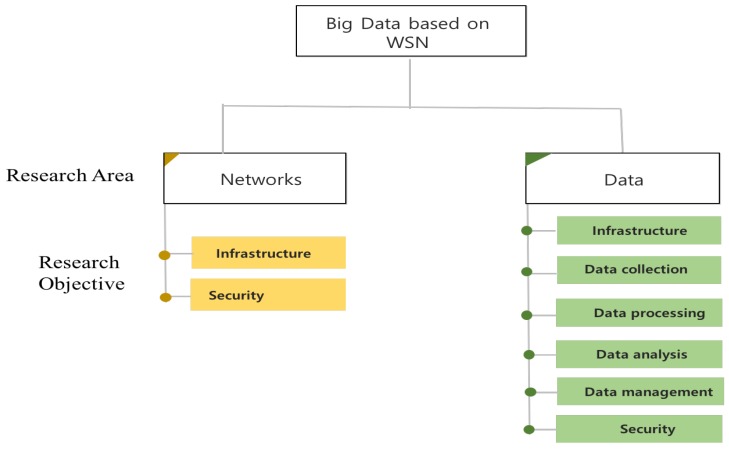
Classification of related work.

Research on big data systems based on WSNs is largely categorized into two main areas (Figure 4): network systems and data systems. The former is focused on network systems that deliver the sensor data to the big data system, while the latter is focused on data processing. Each research area has multiple subcategories according to the research objective. This paper introduces two research objectives for network systems and a large number of research objectives for data systems.

The inclusion criteria were as follows: we consider only studies published in English that simultaneously describe the application and network protocol of WSNs and the data processing technologies for big data systems. Only peer-reviewed journal and conference papers were included in the study, to ensure quality and reliability of information. Studies published before 2014 were excluded. The search identified 70 papers based on these inclusion and exclusion criteria.

### 3.3. Network System

#### 3.3.1. Infrastructure

**Figure 5:**
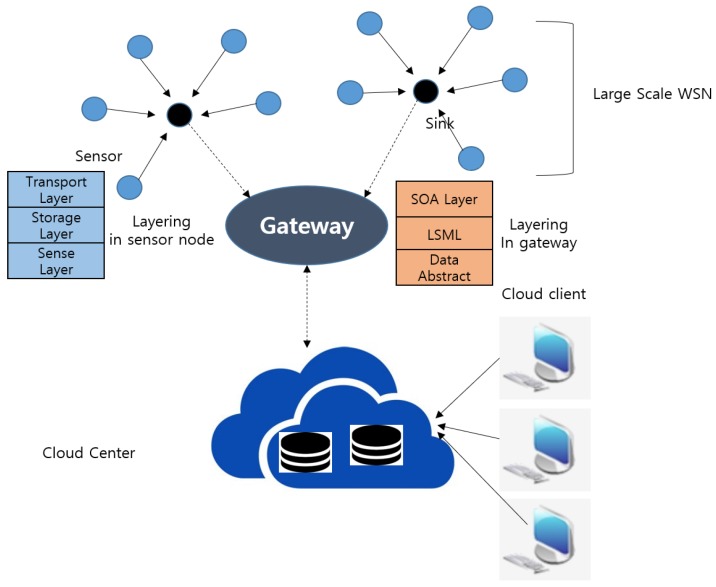
Architecture of SVC4WSN.

In this research area, most work is conducted by proposing either network architectures or communication protocols. First, the authors propose structural construction of the WSN based on big data processing called service-oriented architecture and virtualization cloud for WSN (SVC4WSN) [23] and simulates it numerically through comparison with multi-hop direct forwarding for local wireless sensor network (MDF4LWSN) architecture. The SVC4WSN consists of four layers: a large-scale WSN, gateway, cloud center, and users (as shown in Figure 5). In this architecture, there are two critical issues: congestion caused by the big data, and communication latency. To deal with these issues, flexible and multi-layer data processing and storage models based on cloud computing are proposed.

Another challenging task for gathering big data in a densely distributed sensor network with high energy efficiency is addressed in [24]. The method can be used to determine the sink node’s trajectory and data-gathering through clustering. Unlike the typical clustering scheme, K-medoid clustering in six steps is proposed to keep energy consumption balanced in continuous iterations. Kalnoor et al. [25] proposed a similar approach based on clustering. In their work, the mobility of a sink node was selected as an effective solution to manage big data collection. Moreover, a framework to leverage the correlation between sets of active sensor nodes was presented. Another approach to utilize a mobile data collector is presented in [26], where two different approaches are suggested: data collection using data mule (MULE) and sensor network with mobile access point (SENMA). These approaches are characterized by the number of hops that are required to handle unexpected network partition during mobile data collection. Xu [27] conducted a study that deals with the routing protocol over cluster architecture to deliver multimedia data in WSN. It aims to reduce network congestion, in order to improve the reliability of data transmission, as well as reduce packet loss rate. To achieve these goals simultaneously, an efficient traffic load balancing algorithm is proposed to ensure balanced energy consumption within the network.

In addition to data collection, the data aggregation scheme for big data is discussed in [28] through the information-centric networking (ICN) approach, where data are retrieved by names and in-network caching. The proposed framework operates according to the following steps: (1) the network is initialized and the communication nodes are clustered using low-energy adoptive clustering hierarchy (LEACH) protocol; (2) collected data is aggregated to the cluster head memory; (3) an aggregatable name-based routing (ANBR) method retrieves the data and forwards it to the data center. From another point of view, another architecture for WSN is proposed to prevent excessive energy consumption on a sensor node in case of the high redundancy of sensed data. A framework called structure fidelity data collection (SFDC) [29] leverages the spatial correlations between the nodes by reducing the number of active sensor nodes while maintaining a low structural distortion of the collected data. A node’s duty cycle is controlled by a structural distortion depending on the image quality assessment approach. Thus, the data fidelity in terms of structural similarity in the continuous sensing applications for WSNs can be accomplished by SFDC. In addition, Harb et al. [30] proposed a method of compressing collected data at the first level as well as eliminating redundant data generated by the neighboring node at the second level. The proposed scheme adapts clustering methods (EKmeans and TopK) to decrease the communication costs and enhance data mining in WSN.

As an example of specific-purpose routing protocol, a new routing protocol is proposed to assign a dynamic priority according to the requirements of quality-of-service (QoS) as well as achieve load distribution by involving a larger number of sensor nodes in the path. In particular, high energy efficiency [31] is achieved by selecting a next hop according to the available resources and the required energy cost.

Consequently, the advantages and disadvantages of each method in the research area are compared in Table 1, where energy efficiency and management/maintenance cost are the major metrics.

#### 3.3.2. Security

During data-gathering, WSN performs both data capture and transport, and it is important to accomplish these two tasks in a secure manner. To address this problem, Zhou et al. [32] discuss and analyze several implementations of architecture that aim to provide efficiency and robustness for the internal compromise and external attack. Then, they propose a new architecture: trusted big data capture and transportation. In addition, misleading or forged data-gathering may occur in WSNs. Therefore, sensitive and critical data transmission through secure communication is required. While considering constraints on nodes, symmetric cryptography is very applicable to WSN due to its efficiency. However, symmetric cryptography should work with key management for distribution. To reduce the overhead for distribution, Kandah et al. [33] propose a centralized stateful connection (CSC) that provides efficient key management for dynamic sensor networks. It can maintain a good balance between efficiency and security that is achieved by using public key encryption at the beginning of the node’s life in the WSN. Moreover, security issues for big data in heterogeneous WSN are reviewed and discussed in [34].

### 3.4. Data System

While the network system focuses on delivery of sensed data in WSN, the data system focuses on efficient processing of the data that are transmitted via WSN. The research objectives in data systems are more diverse than in network systems: they include infrastructure, data collection, processing, analysis, management, and security.

#### 3.4.1. Infrastructure

Because of application-specific requirements and usage, manipulation, and exploitation of the data generated by WSN in the relevant data system for big data are in high demand. Thus, multiple research papers address the research challenges related with data systems.

First, an infrastructure may include big data tools for gathering, storage, and analysis of data generated by a WSN that monitors air pollution levels in a city [21]. The proposed framework combines Hadoop and Storm for data processing, storage, and analysis, and Arduino-based kits for constructing unique sensor prototypes. In terms of components, the proposed system is composed of three main modules: data acquisition module (DAM), data processing module (DPM), and a messaging tool between DAM and DPM.

In addition to models of complete systems, there are models of specific components. For example, as for distributed data-centric storage in WSN, Xu et al. [35] propose a big data storage and retrieval algorithm for WSN under nonuniform node distribution. It aims at estimating the additional overhead for the real distribution of sensor nodes. As a technical enhancement, data redundancy among neighbor nodes and a simple routing protocol are proposed and evaluated in the experiments. The main contribution of their work is to reduce the energy consumption and improve the retrieval efficiency with the aid of proximity and routing algorithms, without the aid of GPS. Furthermore, three-tier data mining paradigm for big data in WSN is presented by Yoon [36]. The micro-controller, smartphone, and host server tier are responsible for streamlining the sensor data transmission, forming the patterns in sensor data sets, and providing human expertise associated with the patterns. The proposed architecture leads to low-lost data transmission, early time-critical data mining, and urgent response for medical as well as healthcare applications. Additionally, it is interesting to introduce a smartphone device in the tier. Furthermore, a detailed algorithm and approach for data mining are proposed to achieve the goal.

New requirements for integrating a WSN system and its associated services into a big data system are defined in [37]. To implement this system, a holistic architecture is proposed to consider the flows of the data from sensors. Specifically, a constrained application protocol (CoAP) and a Linux service to integrate Hadoop with HBase datastore are employed over the core architecture on Linux. In the proposed architecture, multiple layers for node possess different capabilities depending on their roles.

In summary, major approaches in this research category are compared in Table 2 in terms of advantages and disadvantages. Because most of these schemes are evaluated only for prototypes, further validation is required.

#### 3.4.2. Data Collection

Although several approaches to data collection were already introduced in the terms of network system infrastructure, different algorithms will be presented in this section. This implies that data collection through static and mobile sink are not changed in the data system. However, new models and procedures are defined according to the requirements of the system.

First, context-aware data mules (CADAMULE) are proposed as a solution for smart data collection in WSN [38]. In this work, Jayaraman et al. extend the context spaces modeling theory to discover new context attributes, by considering previous scenarios and events. This information is used to capture the context information. Moreover, the proposed context-aware data mule aims at delivering the data to the sink. Consequently, another approach to introduce a mobile collector is explained in [39]. This research determines the mobile sink node’s trajectory by introducing an M-mobile collector based on a clustering algorithm. In the proposed scheme, mobile collectors traverse a fixed path to collect data from cluster centroids and sensors in the clusters by multi-hop routing. Zhu et al. [40] proposed a four-phase mobile data collecting protocol, called mobility-assisted big data collecting protocol (MDCP). MCDP operation is largely divided into network clustering, route planning, route combination, and data collecting. During these procedures, mobile sinks need to visit all the source nodes along the constrained routes while minimizing energy consumption. However, because this problem is NP-hard, two heuristic algorithms are proposed, accordingly. The former algorithm builds routes by adding a link to the partially formed routes between two end nodes based on a measure of cost savings, while the latter algorithm follows the sequential route building algorithm. To demonstrate suitability of the proposed scheme, the performance of a mobile collector is evaluated and compared in [41]; their main objective is to evaluate expectation maximization (EM) based clustering scheme as a function of the number of mobile nodes.

In addition to a mobile collector, another main issue of data collection for big data systems is energy efficiency. First, Takaishi et al. [42] adapt sink node’s mobility. A new mobile sink routing and data-gathering method through the network clustering based on a modified expectation-maximization technique is suggested. Additionally, an optimal number of clusters to minimize the energy consumption through connectivity and data request flooding model are derived. Apart from energy efficiency, the problem of a long latency caused by the mobile collector is addressed in [43]. For this problem, Sujithra et al. propose two novel approaches: energy-efficient big data-gathering using local data collector (EEBDG-LC) and energy-efficient big data-gathering using local data collector with threshold (EEBDG-LCWT), to defeat an increased data-gathering latency of the previous algorithm named “toward energy-efficient big data-gathering” (TEEBD). The main issue is in placing a local data collector in every centroid of the region. Further, the amount of traffic is adjusted to prevent overwhelming by the predetermined threshold value. In the aspects of communications protocol, energy-efficient routing protocols to gather real-time data are proposed in [44]. Rani et al. present an energy-efficient big data algorithm called “big data efficient gathering” (BDEG). Cluster header in BDEG is determined depending on the level of both the received signal strength indicator and the residual energy of the sensor nodes. Each cluster header (CH) within a cluster and relay node (RN) is connected to the cluster coordinator (CCO) nodes for inter-cluster communication. The proposed algorithm builds an energy-efficient route by balancing the load on the cluster headers, cluster coordinators, and relay nodes for gathering big data in an effective manner. Finally, adaptive distributed data-gathering (ADiDaG) technique to save energy in periodic WSN applications is proposed in [45]. Depending on operations in every round, ADiDaG takes data-gathering, sampling decision, and transmission phases. The main decision algorithm for each phase depends on the longest common subsequence similarity and grouping approach, as well as adaptive sensor sampling rate. Finally, a special case for data collection in indoor WSN is explained in [46]. Ding et al. propose a real-time big data-gathering (RTBDG) algorithm for the risk analysis of the industrial operations. The proposed algorithm is performed according to the requirements of the risk analysis under the clustering architecture, which is built by using received signal strength indicator and residual energy information. Simulation results are given to demonstrate the suitability of RTBDG for industrial environments.

In summary, Table 3 lists a comparison of data collection schemes in terms of advantages and disadvantages. Even though most of the schemes are well-designed to achieve their objectives, there are still research challenges in the aspects of deployment or optimization.

#### 3.4.3. Data Processing

In this section, we describe in-network processing in WSN: data aggregation and fusion technologies. Fouad et al. [19] provided an overview and discussion of state-of-the-art data mining and data fusion techniques designed for WSNs. They emphasized that processing sensor data inside the network (in-network) before any further processing helps save limited resources and prevent excessive data duplication.

In addition to a comprehensive survey, a specific algorithm called high-dimensional data aggregation control algorithm for big data (HDAC) is proposed in [47]. In this study, information to eliminate the dimension not matching with the specified requirements is the main source. While handling this, the principal components method to analyze the remaining dimension is employed. In the process of data aggregation, the self-adaptive data aggregation mechanism is used to reduce the phenomenon of network delay. The simulation results evaluate node energy consumption and data delay through the HDAC. Recently, a comprehensive survey of data aggregation for big data in WSN was presented in [19]. It covers research challenges in the big data area and proposes a new classification of these challenges, accordingly to the necessities of WSN. The detailed data aggregation strategies in WSNs are also discussed. They include: (1) distributed compressive data aggregation in large-scale WSN; (2) sensor data aggregation in a multi-layer big data framework; (3) lifting wavelet compression based data aggregation in big data WSN; (4) scalable privacy-preserving big data aggregation mechanism; and (5) a cluster-based data fusion technique to analyze big data in wireless multi-sensor systems.

#### 3.4.4. Data Management

There is a book chapter on big data management in WSN edited by Hung and Hsieh [48], where an overview of big data management on WSN is presented. Moreover, big data tools and frameworks are introduced to evaluate performance of query processing and data collection. As the major challenge in data management in WSNs, the authors focus on energy preservation. In the aspects of energy efficiency, the authors emphasize decentralization, which is one of the promising ways to achieve energy preservation by distributing the computation tasks among sensor nodes.

Moreover, Medlej [49] proposes novel big data management techniques for periodic sensor networks to overcome the limitations imposed by WSN as well as adapt key features of sensor data. The main contribution of this thesis is to propose an adaptive sampling approach for periodic data collection by allowing each sensor node to adjust its sampling rates in parallel with the physical changing dynamics. Additionally, periodic data aggregation on sensor node data is proposed as a preprocessing phase for an efficient and scalable data mining. Specifically, a new data mining method based on the existing K-means clustering is proposed.

#### 3.4.5. Data Analysis

Prior to analyzing big data, it is necessary to define system architecture. To meet this requirement, Farrah et al. [20] propose a new framework that consists of Hadoop over MapReduce and Hive data warehouse. To perform MapReduce jobs, an SQL-like language, HiveQL, is used to execute queries. Based on these techniques, a data warehouse model for WSN is proposed to analyze all gathered data and detect abnormal behavior.

As a result of data analysis, fast detection and identification of the location of errors in sensor datasets should be provided. To achieve this goal, Yang et al. [50] developed a novel data error detection approach that exploits the full computational potential of the cloud platform and the network features of WSN. In the proposed scheme, a set of sensor data error types are classified and defined over cluster based WSN. In particular, the error detection over a scale-free network topology and most of the detection operations can be conducted in limited temporal or spatial data blocks instead of the complete big data set. Another error detection, as well as cleaning big data sensor error issues, are addressed in [51]. In this study, the performance is improved by employing a scale-free network topology and classification of the error types at the same time. Moreover, extensible sensor stream processing (ESP) can be used for pervasive applications to build sensor data cleaning infrastructures.

As one of the important applications for WSN, intruder detection becomes pervasive with an accurate measurement of the image in terms of width and resolution. To operate it practically, a three-layered big data analytic architecture [52] is designed to analyze the data generated during the construction of the barrier and the detection of the intruder using the camera sensors. In addition, a cloud layer is implemented to study the behavior of the intruder with a minimum number of camera sensors. As indicated in the previous case, data analysis becomes more beneficial in wireless multimedia sensor networks, compared with typical WSN. Thus, a big data model based on a graph database model that deals with data generated by wireless multimedia sensor networks is proposed in [53]. With the help of a simulator to generate synthetic data, and store and query big data in the proposed model, the authors present the evaluation results for well-known graph-based NoSQL databases.

#### 3.4.6. Security

To detect intrusion efficiently and correctly, a WSN is usually a good solution: it can defend against the insider attacks using a relevant trust-based mechanism. However, due to excessive data, effectiveness of trust computation can be degraded significantly. To prevent this degradation, Meng et al. [54] proposed Bayesian-based trust management with traffic sampling under a hierarchical structure. Their experimental results prove that the proposed approach improved trust management in a high-traffic case by detecting malicious nodes quickly.

Security in multimedia big data applications for smart city in trust-assist sensor cloud (TASC) is addressed in [55]. Two types of TASC: TASC-S (TASC with a single trust value threshold), and TASC-M (TASC with multiple trust value thresholds), are proposed and evaluated through extensive simulation results in the aspect of throughput. Further, Wu et al. [56] proposed a dynamic trust relationships aware data privacy protection (DTRPP) mechanism by combining key distribution with trust management. The major contribution of this work is to evaluate the trust value of a public key according to both the number of supporters and the trust degree of the public key.

## 4. Open Issues

### 4.1. Evolution to Internet-of-Things (IoT)

As described in [57], IoT systems will be employed with typical WSNs and new communication systems. Rather than typical WSN, IoT system [58] exhibit better performance in terms of latency and energy efficiency in real implementations. IoT collects and uses data in real time to optimize operations, detect security breaches, correct malfunctions, and more. Therefore, an IoT system must include managing real-time streaming data, real-time analytics, and real-time decision making. It implies that IoT should have the ability to ingest, aggregate, and compress real-time data from sensor devices at the edge. In this situation, big data systems play a great role in analyzing real-time sensor data and rendering real-time decisions that optimize operational performance or detect unusual performance or behaviors for an immediate investigation. Based on the above perspective, IoT will replace WSN for big data systems. As a good reference model, Marjani et al. [59] propose a new architecture for big IoT data analytics with practical use cases. Furthermore, privacy, big data mining, visualization, and integration are presented as future research directions. However, as indicated in [60], there are emerging issues to be addressed. As a solution to these problems, adaptive real-time response and intelligent control technologies will be required.

### 4.2. Application-Specific Requirements

Usually, WSN is deployed and implemented to accomplish application-specific missions, as well as meet their specific requirements. For example, some applications require scalar values, while others require vector ones. According to the required type of values in the application, the data generated by the sensor node possesses naturally different properties and characteristics. This implies that the network and data system for big data should be designed in accordance with the application requirements. Moreover, because multimedia data need more considerations for resource consumption than typical data in terms of storage, processing, and management, a feasible framework and architecture should be developed to employ specific algorithms regardless of the volume, velocity, and variety of the generated data.

### 4.3. Network Architecture

In terms of WSN for big data systems, mobile sink [61] and clustering [62] are important research challenges in collecting and aggregating the data. However, these schemes need to be extended to significantly reduce energy consumption and provide real-time communications due to a large amount of data. Examples include [63,64] that propose a data aggregation algorithm based on principal component analysis (PCA) to achieve minimal energy consumption and delay, which can be executed in the cluster head (CH).

In addition, as new applications emerge, typical WSN is evolved to support new types of sensor networks: urban sensor networks, flying sensor networks, wireless underground sensor networks, and wireless underwater sensor networks, etc. To handle big data in these new networks, the current network architecture that usually employs either mobile sink or clustering need to be extended or modified to collect the sensed data efficiently in several areas. For example, mobility patterns in these networks are completely different from typical WSN: connectivity between source and destination is not always guaranteed. Thus, feasible mobility prediction and clustering algorithms should be developed according to the additional constraints of each network. Additionally, clustering algorithms need to be revised in the network environment, while considering connectivity and mobility. Moreover, regardless of the type of the network, energy efficiency is the most critical issue to be addressed. Therefore, network architecture and communication protocols need to meet the above requirements. Finally, deployment issues of controlling the topology in new types of WSN should be mentioned. In addition, overall data generated across numerous sensors in the densely distributed WSNs can produce a significant portion of the big data. Thus, a data fusion algorithm needs to be supported, as studied in [65]. The authors introduce data fusion model based on an improved radial basis function (RBF) network. Additionally, different data packets set different transmission intervals and properties according to the dynamics of topology under the clustering architecture.

### 4.4. Real-Time Communications on Fog Computing

**Figure 6:**
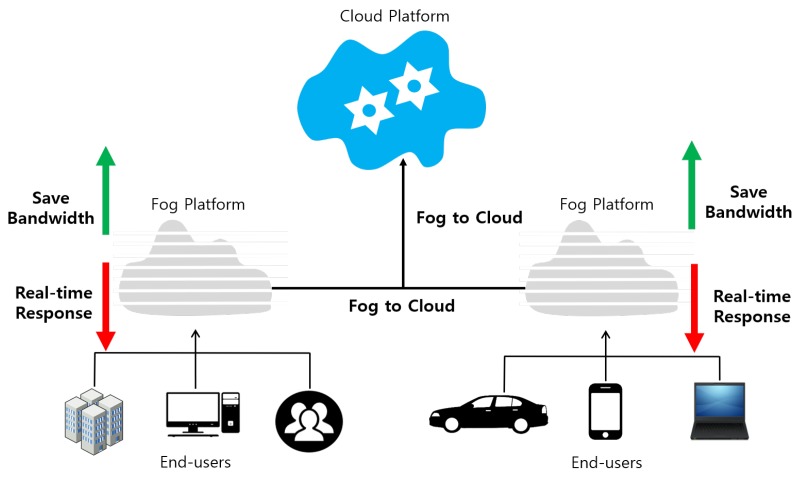
Overview of fog computing.

Because of the delay-sensitive property of sensed data in WSN, real-time communication and processing are highly preferred for big data systems. However, severe constraints on nodes in WSNs make it difficult to support real-time communications. Moreover, a big data system on cloud computing architecture is not suitable to perform real-time tasks. In order to support real-time tasks, fog computing [66] was recently proposed to extend the cloud services to the edge of the network by computation, communication and storage closer to the edge devices and end users, which aims to enhance low latency, mobility, network bandwidth, security and privacy. Therefore, a big data system on fog computing can be a feasible solution to support real-time processing. However, there is no outstanding solution for real-time communications [67] in WSN yet. Figure 6 shows the conceptual architecture for fog computing, which works as an intermediate layer between the cloud and the users to provide real-time communications.

### 4.5. Extensive and Flexible Framework

Even though WSN is regarded as one of the major sources for big data, there are many other types of sources for big data. They are used in applications that are built over radio frequency identification (RFID), machine-to-machine (M2M) communications, peer-to-peer (P2P) communications, internet-of-things, and smartphones. If the data from WSN can be combined with data from other applications, it is possible to build a more useful big data system. This analysis implies that the data from these devices need to be integrated and handled in a homogenous way. In other words, there is a demand in defining an integrated, extensive, and flexible framework to accommodate various types of data. Further, data fusion may be an alternative approach. As for the similar tasks, the research work for middleware has been conducted actively. Therefore, it is recommended to analyze the state-of-the-art of middleware for WSN [68] and develop a feasible framework to perform integration of several data types.

### 4.6. Modeling and Simulation

Performance evaluation and comparison are essential in research work. Even though some platforms are proposed for big data and WSN, respectively, there is no integrated and combined simulator or testbed. As a short-term solution, it seems reasonable to include a WSN simulation into a big data simulator or platform for extensive experiments. On the other hand, for a long-term solution, it is required to develop an interface between two platforms to exchange data in a way of eliminating dependency. Moreover, as mentioned before, there is also demand in building a flexible and extensible evaluation platform to include diverse types of WSNs. Additionally, because evaluation is mostly dependent on the modeling, more realistic traffic and error modeling is critical to improve the reliability of the simulation.

In summary, Table 4 shows the current research challenges and further directions for big data systems based on WSN.

## 5. Conclusions

In this paper, we presented technical research challenges for big data systems based on WSN in cases when WSN was regarded as one of the major data sources. Prior to looking into major contributions of each work, we presented the opportunities of big data systems in WSN. Subsequently, previous research was categorized according to the research area and objective. Specifically, we focus on data collection and in-network processing while considering the unique characteristics of network properties. Finally, open issues in this research area are discussed.

## Figures and Tables

**Table 1:** Comparison of advantages and disadvantages for infrastructure.

Method	Key Feature	Advantage	Disadvantage
SWV4WSN [17]	Multi-layer model	Good lifecycle	High management cost
[18]	K-medoid clustering	High energy efficiency	High maintenance cost
[20]	Mobile sinks	Good for network partition	High energy consumption in few clusters
[21]	QoS provisioning	Balanced energy consumption	High complexity
[22]	Name and in-network caching	High energy efficiency	Low energy consumption in many clusters
[23]	Spatial correlation	High energy efficiency	No consideration for temporal correlation

**Table 2:** Comparison of advantages and disadvantages of data systems.

Method	Key Feature	Advantage	Disadvantage
[28]	Framework based on Hadoop and Storm	Open-source based implementation	Low reliability
[29]	Distributed data-centric storage	High energy efficiency	Deployment issues in real-world scenarios
[30]	Three-tier data mining	Urgent response	Experiments in few scenarios

**Table 3:** Comparison of advantages and disadvantage for data collection.

Method	Key Feature	Advantage	Disadvantage
CADAMULE [32]	Situation and event awareness	Cost-efficient data collection	Simple weight based computation
[33]	M-mobile collector	High energy efficiency	Few scenarios for simulation
[34]	MDCP	High energy efficiency	Assumption of infinite storage memory
[36]	Mobile collector	High energy efficiency	Flooding based operation
[37]	Local data collector	Reduced data-gathering latency	Too much dependency on threshold value

**Table 4:** Summary of open issues.

Research Area	Current Research Challenges	Further Trends
Networks Architecture	Static/mobile sink, aggregation	Energy efficiency, Specific protocols for applications, 5G Communications
Framework	Single platform for WSN	Integrated platform for M2M, P2P, and IoT
Real-time	QoS protocol	Fog/Edge computing, Middleware
